# RWRNET: A Gene Regulatory Network Inference Algorithm Using Random Walk With Restart

**DOI:** 10.3389/fgene.2020.591461

**Published:** 2020-09-25

**Authors:** Wei Liu, Xingen Sun, Li Peng, Lili Zhou, Hui Lin, Yi Jiang

**Affiliations:** ^1^School of Computer Science, Xiangtan University, Xiangtan, China; ^2^Key Laboratory of Intelligent Computing and Information Processing of Ministry of Education, Xiangtan University, Xiangtan, China; ^3^School of Computer Science and Engineering, Hunan University of Science and Technology, Xiangtan, China

**Keywords:** gene regulatory networks, random walk with restart, local topology, global topology, Markov Blanket discovery algorithm

## Abstract

Inferring gene regulatory networks from expression data is essential in identifying complex regulatory relationships among genes and revealing the mechanism of certain diseases. Various computation methods have been developed for inferring gene regulatory networks. However, these methods focus on the local topology of the network rather than on the global topology. From network optimisation standpoint, emphasising the global topology of the network also reduces redundant regulatory relationships. In this study, we propose a novel network inference algorithm using Random Walk with Restart (RWRNET) that combines local and global topology relationships. The method first captures the local topology through three elements of random walk and then combines the local topology with the global topology by Random Walk with Restart. The Markov Blanket discovery algorithm is then used to deal with isolated genes. The proposed method is compared with several state-of-the-art methods on the basis of six benchmark datasets. Experimental results demonstrated the effectiveness of the proposed method.

## Introduction

Inferring accurate gene regulatory networks (GRNs) is an exciting but difficult topic in the field of bioinformatics. Inferring accurate GRNs is not only helpful to understanding complex regulatory relationships between genes in cells but also to understanding relationships between genes and diseases (Lv and Bao, 2009; Altay and Emmert-Streib, 2010; Tang et al., 2015). With the development of high-throughput technologies, huge gene expression data have been produced from which researchers can infer GRNs (Maetschke et al., 2014; Liu, 2015).

Numerous network inference methods for inferring accurate GRNs have been developed. These methods can be classified into two categories: model-based and similarity-based methods. Model-based methods, which mainly include Boolean network model, differential equation model and Bayesian network model, usually infer GRNs through a computational model. The Boolean network model is a simple discrete model that contributes to understanding various states of cells, such as proliferation, differentiation and apoptosis (Huang, 1999; Lim et al., 2016; Zhou et al., 2016). However, the Boolean network model cannot be applied in networks with complex regulatory relationships. The differential equation model is a continuous network model that can accurately describe the dynamic characteristics of GRNs. The expression level of genes in differential equation is determined by related genes and regulatory equations, thus allowing the underlying phenomena of organisms to be accurately described (Alter et al., 2000; Cantone et al., 2009; Honkela et al., 2010; Huppenkothen et al., 2017). The Bayesian network model is a popular graphical model of probability. In this model, the dependencies between genes are described by a directed acyclic graph. The Bayesian network model is superior to other models in terms of dealing with noise and prior knowledge, but it has high computational complexity (Tan et al., 2011; Betliński and Ślęzak, 2012; Shi et al., 2016).

Similarity-based methods, which primarily include correlation-based and information theory-based methods, identify regulatory relationships by measuring the dependencies between genes (Li et al., 2011). In correlation-based methods, the dependencies are determined by the degree of co-expression. Typical measurement methods include Pearson’s correlation coefficient, Euclidean distance and partial correlation coefficient (de la Fuente et al., 2004; Saito et al., 2011; Fukushima, 2013; Ruyssinck et al., 2014; Mohamed Salleh et al., 2015; Ghosh and Barman, 2016). However, these measurement methods cannot identify complex dependencies, such as non-linear dependencies (Wang and Huang, 2014). Information theory-based methods can capture complex non-linear regulatory relationships (Brunel et al., 2010; Mousavian et al., 2016). Mutual information (MI) is first used in information theory to measure the similarity between signals and later used in the field of biology to measure regulatory relationships between genes. Classical methods include Relevance Network (RN), Minimum Redundancy Network (MRNET), Path Consistency Algorithm based on Conditional Mutual Information (PCA-CMI) and Redundancy Reduction in the MRNET algorithm (RRMRNET). RN (Butte and Kohane, 2000; Kuzmanovski et al., 2018) is one of the earliest methods that used MI to measure relationships. MRNET (Meyer et al., 2008) is a feature selection method. In MRNET, a feature selection strategy is adopted in selecting regulatory relationships. Although non-linear regulatory relationships can be measured by MI, it cannot distinguish indirect regulatory relationships (Margolin et al., 2006). To overcome this limitation, Zhang et al. (2012) proposed PCA-CMI, in which MI is replaced by conditional mutual information (CMI). However, CMI tends to underestimate the relationship between genes, so Zhang et al. (2015) proposed conditional mutual inclusive information (CMI2) to solve the problem of underestimation of CMI. To improve accuracy, Liu et al. (2017) proposed RRMRNET on the basis of MRNET, in which two strategies are implemented in eliminating redundant regulatory relationships.

In addition, several machine learning-based methods, such as tree-based ensemble regression and neural network-based inference methods, have been applied in this field (Huynh-Thu et al., 2010; Huynh-Thu and Sanguinetti, 2015; Petralia et al., 2015; Raza and Alam, 2016). Researchers have also noticed that several regulatory relationships do not occur in every cell. Thus, the GRN should be defined in specific cells and situations (Moignard et al., 2015; Moris et al., 2016). Therefore, network inference methods based on single-cell expression data have attracted people’s interest, which has led to the development of computational and statistical methods that are aimed at discovering new insights into cell state transitions (Bendall et al., 2014; Trapnell et al., 2014; Pina et al., 2015; Rue and Martinez Arias, 2015). The use of single-cell expression data to infer networks has many advantages. With the development of single-cell technology, the amount of data we can use will increase, which can effectively alleviate the defects of high-dimensional and low-sample gene expression data (Macosko et al., 2015). However, obtaining the time-series data of single cells is currently impossible. Notably, these methods infer the regulatory relationship based on the similarity between the transcriptional states of genes and usually provide strong assumptions, which are often unconvincing. However, several methods can still be used for network reasoning using single-cell expression data (Bendall et al., 2014; Trapnell et al., 2014; Haghverdi et al., 2016; Moris et al., 2016; Reid and Wernisch, 2016).

Although these aforementioned methods have extensively promoted GRN research, they still have certain shortcomings. For example, model-based methods usually have high computational complexity. Most similarity-based methods consider relationships between only two and not all genes at a time. Moreover, these methods usually focus on the surrounding information rather than on the global topology of network, thus resulting in numerous redundant regulatory relationships. Therefore, the present study mainly concentrates on inferring GRNs by combining local and global topologies.

Random Walk with Restart (RWR) is an improvement of the Random Walk (RW). RWR is widely used in the field of bioinformatics because it can capture multivariate relationships between nodes and explores the global topology of networks (Rosvall and Bergstrom, 2008; Athanasiadis et al., 2017; Peng et al., 2018; Valdeolivas et al., 2019). Chen et al. (2012) used RWR to determine associations between diseases and miRNAs. Sun et al. (2014) verified the robustness of RWR for parameter selection. Luo et al. (2016) proposed a new computational approach, MBiRW, that uses a combination of similarity measures and a double random Walk (BiRW) algorithm to identify potential new indications for a particular drug. Yu et al. (2017) provided a comprehensive framework for predicting new HCC drugs based on multi-source random walk.

To address the limitations in gene network inference, we propose a novel network inference algorithm using RWR (RWRNET). The restart probability, initial probability vector and roaming network in RWR is first improved to apply it in network inference. Second, the improved RWR is used in inferring network structure. Finally, the Markov–Blanket discovery algorithm IPC-MB is used to optimise the network structure to obtain the final gene network. The main contributions of this study are described as follows:

(1) We improve the three key elements of RWR. First, the proposed method obtains the restart probability and initial probability vector according to node connectivity and functional modularity and then captures the local topology structure of the network. Second, a roaming network construction method is proposed for reducing the complexity of regulatory relationships among genes.
(2) We use a Markov–Blanket discovery algorithm (IPC-MB) to deal with isolated genes in the network that are generated by the RWR process.
(3) Extensive experiments are conducted to evaluate the performance of RWRNET. Experimental results confirmed that RWRNET is an effective network inference method.

## Theory

In this section, we review the concepts of (conditional) mutual information, RWR and Markov–Blanket that are related to the proposed method.

### (Conditional) Mutual Information

Mutual information is an information measurement in information theory. MI can be regarded as the information shared by two random variables or the reduction of uncertainty due to a known random variable. The MI between random variable *X* and *Y* is defined as follows:

(1) $$\mathrm{MI}\left(X,Y\right)=\sum_{x\in X,y\in Y}p\left(x,y\right)\log\frac{p\left(x,y\right)}{p\left(x\right)p\left(y\right)}\;$$

where *p*(*x*,*y*) is the joint distribution of *X* and *Y*; while *p*(*X*) and *p*(*Y*) represent the marginal probability functions of *X* and *Y*, respectively.

Conditional mutual information (CMI) is a variant of MI. CMI represents the information shared between variable *X* and variable *Y* under the influence of variable *Z*. The CMI between variable *X* and variable *Y* is defined as follows:

(2) $$\mathrm{CMI}\left(X,Y|Z\right)=\sum_{x\in X,y\in Y,z\in Z}p\left(x,y,z\right)\log\frac{p\left(x,y|z\right)}{p\left(x|z\right)p\left(y|z\right)}\;$$

where *p*(*x*,*y*,*z*) is the joint distribution of *X*, *Y* and *Z*, *p*(*x*|*z*) is the marginal distribution of variable *X* when variable *Z* occurs; and *p*(*x*,*y*,*z*) is the joint distribution of *X*, *Y* under the influence of variable *Z*.

### Random Walk With Restart

Random Walk with Restart is an improvement of RW. RWR contains a parameter α as the restart probability, and 1 − α represents the probability of a walker moves from a node to an adjacent node. The RWR of graph can be defined by assigning a transition probability to each edge. In this way, a walker can jump from one node to another, and the sequence of nodes visited by the walker is called RWR. Let *p*_t + 1_(*j*) denote the probability that walker locates at *j*-th node when it come to a stable state, then the formula is:

(3) $$p_{t+1}=\left(1-\alpha \right){\mathrm{Wp}}_{t}+\alpha p_{0}$$

where *W* = [*a*_ij_]_N×N_ is the transition probability matrix, *a*_ij_ is the transition probability from the *i*-th node to the *j*-th node; and *p*_*0*_ represents the initial probability vector of *N*×1, in which the *i*-th element is 1 and the others are zero. *N* is the number of nodes in the graph.

### Markov–Blanket

This section introduces Markov-Blanket (MB). In the complete set *U* of random variables, for a given variable *X* ∈ *U* and variable set *MB* ∈ *U* (*X*∉*MB*), the following exists:

(4) X⊥{U-MB-{X}}|MB

that is, if the variable *X* and the set {U-MB−{*X*}} are independent of each other under *MB*, then the minimum variable set *MB* that can meet the above conditions is called *MB* of *X*.

## Methods

**FIGURE 1:**
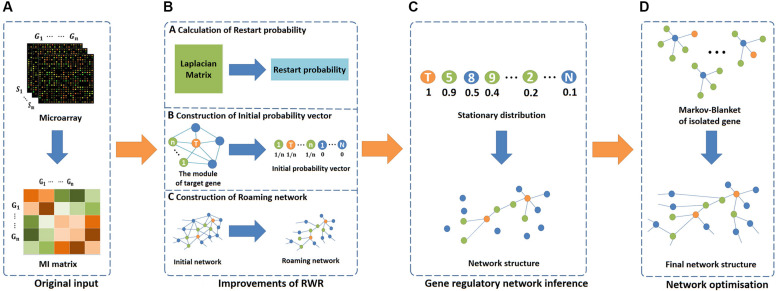
Flowchart of RWRNET. This flowchart consists of four parts, namely, the original input stage, algorithm improvement stage, network inference stage and network optimisation stage. **(A)** In the original input stage, the MI matrix was obtained through gene microarrays data. **(B)** In the algorithm improvement stage, three key elements of RWR were improved. The first was the restart probability, which was calculated by the pseudo inverse of the Laplacian matrix. Then, the initial probability vector was improved next. Different genes have various initial probability vectors, depending on the functional module. In the figure, the centres of modules are represented by orange nodes, modular genes are indicated by green nodes, and others are denoted by blue nodes. Finally, the roaming network. In this paper, the network represented by the mutual information matrix was considered a fully connected network, and the roaming network was obtained by adjusting this network. **(C)** In the network inference stage, RWR was executed to obtain a stationary distribution, and the gene regulatory network was inferred based on the stationary distribution. **(D)** In the network optimisation stage, the MB for each isolated gene was discovered to establish a relationship between the isolated genes and others.

In this study, we propose an effective network inference method (i.e., RWRNET). To apply RWR in GRNs, we improve its three key elements, namely, restart probability, initial probability vector and roaming network. Then the RWR is used to infer network structure. Finally, we use IPC-MB to optimise the network structure. Figure 1 presents the flowchart of RWRNET. Specific details are discussed in the following sections. At the same time, we have uploaded the source code (MATLAB format) to the Internet, and readers can view it by visiting the link^1^.

### Improvements of RWR

This section mainly introduces specific improvements to the three elements of RWR (i.e., restart probability, initial probability vector and roaming network) when RWR is applied in GRNs. First, the restart probability and initial probability vector are determined according to node connectivity and functional modularity to capture the network topology. Second, a roaming network is constructed using the asymmetric MI ranking strategy to reduce the complexity of regulatory relationships among genes. Specific details are described as follows.

#### Calculation of Restart Probability

Different nodes in a network have different connectivity, which reflects network topology structure to some extent. Laplacian Eigenmaps is an effective way to obtain network topology, because it can map high-dimensional data to low-dimensional data and ensure their similarity to the original data as much as possible. Applying discrete Laplacian Eigenmaps to the graph network can obtain the Laplacian matrix *L*. And the pseudo inverse *L*^+^ of *L* is a valid kernel that can provides a similarity measure between nodes. On the basis of *L*^+^, the average commute time ACT (*g*_i_,*g*_j_) between gene *g*_i_ and gene *g*_j_ can be then defined as

(5) $$\mathrm{ACT}\left(g_{i},g_{j}\right)=L^{+}\left(g_{i},g_{i}\right)+L^{+}\left(g_{j},g_{j}\right)-2L^{+}\left(g_{i},g_{j}\right)$$

(6) $$L=D^{-\frac{1}{2}}\left(D-W\right)D^{-\frac{1}{2}}$$

where *W* is the adjacency matrix of graph, which is MI matrix in this paper; and *D* = *diag*(*a*_i_.) with $d_{\text{ii}}={\left[D\right]}_{\text{ii}}=a_{\text{i}}.=\sum_{j=1}^{n}a_{\text{ij}}$; *ACT*(*g*_i_,*g*_j_) describes the average number of steps that particles moves from *g*_i_ to *g*_j_ and then back to *g*_i_.

The average commute time increases when the number of paths connecting the two points increases and when the length of paths decreases. According to this idea, the average commute frequency *ACF*(*g*_i_,*g*_j_) and restart probability α can be defined as follows:

(7) $$ACF(g_{i},g_{j})=\left\{ \begin{array}{c} \begin{array}{ccc} 1 & , & g_{i}=g_{j}\\ \frac{1}{ACT(g_{i}} & , & g_{j}),g_{i}\neq g_{j} \end{array} \end{array}\right.$$

(8) $$\alpha =\frac{1}{N^{2}}\sum_{g_{i}\in G}\sum_{g_{j}\in \text{G}}\text{ACF}\left({\text{g}}_{\text{i}},{\text{g}}_{\text{j}}\right)$$

where *G* = {*g*_1_,*g*_2_,⋯,*g*_N_} is the set of genes, and *N* denotes the number of genes.

#### Construction of Initial Probability Vector

Gene regulatory networks is scale-free network in which only a few genes have regulatory relationships with numerous genes. These genes have substantial expression levels and form their own modules according to different functions. Genes in the same module are closely related not only to each other but also to genes in other modules. In addition, although RWR can obtain the global information of the network, taking only these genes as starting nodes is insufficient. Therefore, the functional module of these genes is used as starting nodes to obtain sufficient information in this paper.

In this study, the sum of MI between one gene and another is used to represent its expression level. The genes whose expression level is higher than the average expression level are selected as the centre of functional module. At the same time, due to the influence of noise on gene expression data, genes with low expression levels less than *MEAN*(*E*L)−*STD*(*E*L) are also selected to fully consider the surrounding information. These genes then put together to form a set *C* that includes not only the genes with high expression levels but also genes with abnormally low expression levels. The expression level *EL* and the set *C* are defined as follows:

(9) $$\mathrm{EL}\left(g_{i}\right)=\sum_{g_{j}\in \left\{G-g_{i}\right\}}\mathrm{MI}\left(g_{i},g_{j}\right)$$

(10) $$\begin{array}{c} C=\left\{g_{i}|\mathrm{EL}\left(g_{i}\right)>\mathrm{MEAN}\left(\mathrm{EL}\right)\mathrm{or}\mathrm{EL}\left(g_{i}\right)\right\}\\ \;\left\{<\mathrm{MEAN}\left(\mathrm{EL}\right)-\mathrm{STD}\left(\mathrm{EL}\right),g_{i}\in G\right\} \end{array}$$

where *MEAN*(*EL*) is the average expression level, and *STD*(*E*L) represents the standard deviation.

Finally, for each gene *g*_i_ in the set *C*, the top *log*⁡*n* genes with the largest *MI*(*g*_i_,*g*_j_) are selected as the functional module *module*_*g_i_*_. Based on these modules, the initial probability vector *p*_*0*_ can be constructed according to the following strategy: for each gene *g*_i_ in *G*, if *g*_i_ is an element of *C*, then the elements of g_i_-corresponding and module-corresponding have a value of non-zero, with their sum equals to 1. Otherwise, only *g*_i_ -corresponding is 1, whereas the others are zero.

#### Construction of Roaming Network

Although GRN is sparse, the regulatory relationships among genes are extremely complicated. Therefore, several classical methods have introduced redundant regulatory relationships when inferring network structure. To reduce the complexity of regulatory relationships while maintaining the local topology, we propose a novel method for constructing the roaming network. The basic idea is to use the asymmetry of MI ranking to adjust the relationships between genes, thereby weakening those that are not closely related. The roaming network (i.e., transition probability matrix) *W* can be constructed using the following formulas:

(11) W(gi,gj)=RankgigjMI*(gi,gj)

(12) $${\mathrm{Rank}}_{g_{i}g_{j}}=\{\begin{array}{c} 1,ifMI\left(g_{i},g_{j}\right)\geq \bar{{\mathrm{MI}}_{g_{i}}}\;\\ 1-\frac{R_{g_{i}g_{j}}}{N},ifMI\left(g_{i},g_{j}\right)<\bar{{\mathrm{MI}}_{g_{i}}}andMI\left(g_{j},g_{i}\right)\geq \bar{{\mathrm{MI}}_{g_{j}}}\\ 0.1,ifMI\left(g_{i},g_{j}\right)<\bar{{\mathrm{MI}}_{g_{i}}}andMI\left(g_{j},g_{i}\right)<\bar{{\mathrm{MI}}_{g_{j}}} \end{array}$$

where *Rank*_*g_i_g_j_*_ is the attenuation factor, which represents the attenuation degree of regulatory relationships; *R*_*g_i_g_j_*_ is the MI ranking of *g*_j_ among the genes connected with *g*_i_. $\bar{MI_{g_{\text{i}}}}$ represents the average MI between gene *g*_i_ and others. As depicted by the formulas, the regulatory relationship between *g*_i_ and *g*_j_ is determined by *R*_*g_i_g_j_*_ when $MI\left(g_{\text{i}},g_{\text{j}}\right)<\bar{MI_{g_{\text{i}}}}$ and $MI\left(g_{\text{j}},g_{\text{i}}\right)\geq \bar{MI_{g_{\text{j}}}}$. The lower the ranking, the higher the attenuation degree will be. If $MI\left(g_{\text{i}},g_{\text{j}}\right)\geq \bar{MI_{g_{\text{i}}}}$, the regulatory relationship between *g*_i_ and *g*_j_ will not be weakened; if $MI\left(g_{\text{i}},g_{\text{j}}\right)<\bar{MI_{g_{\text{i}}}}$ and $MI\left(g_{\text{j}},g_{\text{i}}\right)<\bar{MI_{g_{\text{j}}}}$, the relationship between them will be weakened by 0.1 times.

### Gene Regulatory Network Inference Based on RWR

This section covers network inference on RWR. Specific details are discussed below.

The first stage involves initialisation of regulatory relationships. In this stage, we obtain the MI matrix *MI*_ij_)_N×N_ from the gene microarrays expression data that contain *N* genes and *M* samples. This matrix is then taken as the input of the method.

The second stage entails implementation of RWR. Given the restart probability, normalised transition probability matrix and appropriate initial probability vector, RWR can be performed on the roaming network for each gene *g*_i_ to obtain the stationary distribution *p*_t+1_. Considering time efficiency and accuracy, when |*p*_t + 1_−*p*_t_| < 10^−6^, *p*_t + 1_ is stable, $p_{t+1}^{\left(g_{\text{i}}\right)}\left(g_{\text{j}}\right)$ represents the probability that *g*_i_ finds *g*_j_.

The final stage concerns GRNs inference. In this stage, stationary distribution is multiplied to transition probability to obtain the final score *MIP*^(*g*_i_)^(*g*_j_) :

(13) MIP(gi)(gj)=pt+1(gi)(gj)W*(gi,gj)   

Based on the final score, GRNs can be inferred according to the following formula:

(14) $$\mathrm{NETWORK}\left(g_{i},g_{j}\right)=\{\begin{array}{c} 1,if{\mathrm{MIP}}^{\left(g_{i}\right)}\left(g_{j}\right)>Threshold\left(g_{i}\right)\\ 0,otherwise \end{array}$$

(15) $$\mathrm{Threshold}\left(g_{i}\right)=\frac{3\alpha }{4}\sum_{g_{j}\in G}{\mathrm{MIP}}^{\left(g_{i}\right)}\left(g_{j}\right)$$

where *NETWORK*(*g*_i_,*g*_j_) represents the regulatory relationship between *g*_i_ and *g*_j_; *Threshold*(*g*_i_) is an adaptive threshold for *g*_i_. In this paper, the threshold of each gene is automatically determined by its prediction results based on the following reasons. The prediction results of each gene were obtained by executing the RWR with different initial probability vectors, and different amounts of information were generated by each execution of RWR. Therefore, the prediction results obtained from different genes cannot be compared and cannot be processed with a fixed threshold. To this end, Eq. 15 was designed to screen the regulatory relationships for each gene. ∑*MIP*^(*g*_i_)^(*g*_j_) was selected as the major component of formula to simultaneously consider the effect of the predicted relationship between all genes and the target gene on the results. However, the regulatory relationship cannot be screened out if only one major component is used. Therefore, we added a factor of 3α/4, which represents the information occupancy of the target gene. Equation 15 indicates that only when the predictive relationship between a gene and the target gene exceeds the total information that the target gene holds can the real regulatory relationship between them be considered.

### Network Optimisation Based on IPC-MB

Given that each gene in GRNs has a unique role, no gene should be isolated. However, RWR cannot handle isolated nodes. Therefore, the isolated nodes are processed by a Markov–Blanket discovery algorithm (IPC-MB) to optimise the network structure. IPC-MB is a classical feature selection algorithm (Fu and Desmarais, 2008). Its main idea is involves eliminating redundant and irrelevant regulatory relationships according to conditional independence to find genes that have direct regulatory relationships with the target gene, CMI stands for the conditional independence in this article. The basic idea is look for a parent–child set (PC) and a spouse set. These sets are then merged to obtain the Markov–Blanket (MB) of the target gene. However, since the genes in the spouse set are actually redundant, we will not use all of Markov-Blanket, but only use the parent-child set (PC). Finally, on the basis of PC, the regulatory relationships between isolated genes and genes in the PC are established to obtain optimised GRNs.

To describe the proposed method comprehensively, Table 1 summarises the complete RWRNET. As shown in the table, Lines 2–10 of the pseudo code are the improvements of RWR, including calculating the restart probability, construction of a roaming network and search for functional modules to construct the initial probability vector. In Lines 11–16, RWR was used to infer the initial network structure. The 17th line was used in IPC-MB to optimise the network structure.

**TABLE 1:** Gene Regulatory Network Inference Algorithm Using Random Walk with Restart.

**Algorithm:** RWRNET
**Input:** Gene microarrays data *G* = {*g*_1_,⋯,*g*_*N*_}
**Output:** A gene regulatory network
1: Construct a MI matrix *MI* according to Eq. 1;
2: Calculate restart probability α using Eq. 8;
3: Construct transition probability matrix *W* using Eq. 11;
4: Calculate gene expression level *EL*_(*g*_*i*_)_ for each gene using Eq. 9;
5: Select centres of functional module and put them into set *C* according to Eq. 10;
6: Construct functional modules: *module*_*g*_1__ = {*g*_1_}, *module*_*g*_2__ = {*g*_2_}, ⋯, *module*_*g*_*N*__ = {*g*_*N*_};
7: For each gene *g*_*i*_ ∈ *C* do
8: Rank the genes *g*_*j*_ in {*G*−*g*_*i*_} according to *MI*(*g*_*i*_, *g*_*j*_) in descending order to form ranking list *MIL*;
9: *module_g_i__* ← the top *log*⁡*N* genes in *MIL*;
10: End For
11: For each gene *g*_*i*_ ∈ *G* do
12: Construct initial probability vector $p_{0}^{\left(g_{i}\right)}$ according to *module*_*g*_*i*__;
13: $p_{t+1}^{\left(g_{i}\right)}=\mathrm{RWR}\left(\alpha ,W,p_{0}^{\left(g_{i}\right)}\right)$;
14: Calculate final score *MIP*^(*g*_*i*_)^ according to Eq. 13;
15: End For
16: Infer network using Eq. 14;
17: Process isolated genes based on IPC-MB;
18: Return the optimised gene regulatory network.

## Experiment

In this section, we introduce the datasets and evaluation metrics used to evaluate RWRNET performance. In the experiment, the performance of RWRNET was compared with that of different methods, namely, CLR, ARACNE, MRNET, MIDER, MI3, MRMSn, PCA-CMI, and RRMRNET, based on information theory. Among these methods, MI3 and MIDER can infer regulatory directions. However, RWRNET does not infer regulatory directions. Hence, we ignored the regulatory direction during the comparisons.

### Datasets

During the experiment, the proposed and other methods were tested and compared in terms of six datasets. The test datasets were divided into simulated and real data, which included the reaction chain data, DREAM3 yeast gene expression data and SOS data. The reaction chain data were downloaded from the KEGG database^2^. The reaction chain data were time-series data. The DREAM3 yeast gene expression data were downloaded from the DREAM3 challenge project^3^. The DREAM3 challenge project provided three types of data; the null-mutant gene knockout data were selected in this article. The SOS data were downloaded from E. coli database^4^. The SOS data were interference data, that is, the measurement data obtained through a series of transcription interference. Table 2 provides a summary of the details of the above six datasets.

**TABLE 2:** Descriptions of the datasets in our experiments.

Datasets	Variables	Samples	Type	Network nodes	Network edges
Reaction chain with four species	4	100	Simulated	4	3
Reaction chain with eight species	8	250	Simulated	8	7
DREAM3-10 genes	10	10	Simulated	10	10
DREAM3-50 genes	50	50	Simulated	50	77
DREAM3-100 genes	100	100	Simulated	100	166
SOS	9	9	Real	9	24

The reaction chain with four species datasets comes from a small linear chain of chemical reactions (Samoilov, 1997). The dataset contained four variables, each of which contained 100 samples. The real network of the reaction chain included of four nodes and three edges.

The reaction chain with eight species datasets comes from a small linear chain of chemical reactions (Samoilov et al., 2001). The dataset contained eight variables, each of which contained 250 samples. The real network of the reaction chain included of eight nodes and seven edges.

The Dream3-10 gene dataset is from a yeast network in DREAM3 (Marbach et al., 2010). The dataset contained 10 genes, each of which contained 10 samples. The corresponding real network structure included of 10 nodes and 10 edges.

The Dream3-50 gene dataset is from a yeast network in DREAM3 (Marbach et al., 2010). The dataset contained 50 genes, each of which contained 50 samples. The corresponding real network structure included 50 nodes and 50 edges.

The Dream3-100 gene dataset is also from a yeast network in DREAM3 (Margolin et al., 2006). The dataset contained 100 genes, each of which contained 100 samples. The corresponding real network structure included 100 nodes and 166 edges.

The SOS dataset is from an SOS network (Ronen et al., 2002). The dataset contained nine genes, each of which contained nine samples. The corresponding real network structure included nine nodes and 24 edges.

### Evaluation Metrics

To verify the effectiveness of the proposed method, we used four evaluation metrics: true positive rate (TPR), false positive rate (FPR), positive predictive value (PPV) and accuracy rate (ACC). TP, FP, TN and FN denote the number of true positives, false positives, true negatives and false negatives, respectively. These four evaluation metrics are calculated as follows:

(16) $$\mathrm{TPR}=\frac{\mathrm{TP}}{\mathrm{TP}+\mathrm{FN}}$$

(17) $$\mathrm{FPR}=\frac{\mathrm{FP}}{\mathrm{FP}+\mathrm{TN}}$$

(18) $$\mathrm{PPV}=\frac{\mathrm{TP}}{\mathrm{TP}+\mathrm{FP}}$$

(19) $$\mathrm{ACC}=\frac{\mathrm{TP}+\mathrm{TN}}{\mathrm{TP}+\mathrm{FP}+\mathrm{TN}+\mathrm{FN}}$$

## Results

### Results of the Chain Structure Network

To verify whether the proposed method has an effect on special networks, such as chain structure network, we selected the expression data of chain structure network with sizes of four and eight as the test datasets.

First, we tested the proposed method on the chain structure network with a size of four. Table 3 shows the performance of RWRNET and other methods in this dataset. Like most methods, RWRNET achieved perfect performance (PPV = 1, ACC = 1) in this dataset.

**TABLE 3:** Comparison of the different methods’ performances in the reaction chain with four species dataset.

	TP	FP	TPR	FPR	PPV	ACC
CLR	3	0	1	0	1	1
ARACNE	3	0	1	0	1	1
MRNET	3	1	1	0.33	0.75	0.833
MI3	2	3	0.667	1	0.4	0.333
MIDER	3	0	1	0	1	1
MRMSn	3	0	1	0	1	1
RRMRNET	3	0	1	0	1	1
PCA-CMI	3	1	1	0.333	0.75	0.833
RWRNET	3	0	1	0	1	1

**FIGURE 2:**
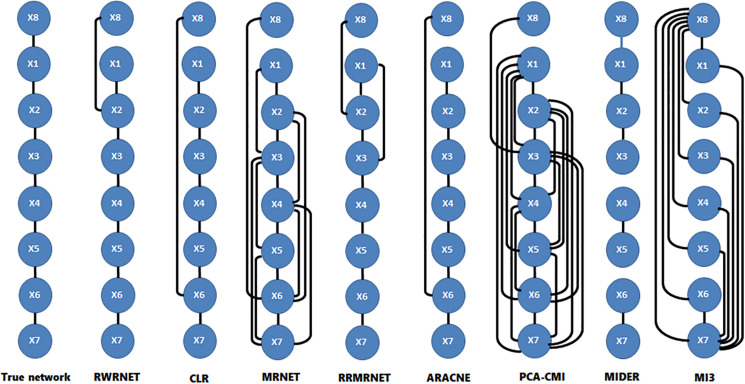
Comparison of the different methods in the reaction chain with eight species dataset.

To verify further the effectiveness of the proposed method, we selected a chain structure network with a size of eight for testing. Table 4 shows the performance of all methods. RWRNET, CLR and ARACNE predicted six correct regulatory relationships (TP = 6), only one missing regulatory relationship and one redundant regulatory relationship (FP = 1). Compared with the performance of the other methods, RWRNET predicted the most regulatory relationships, and its FPR performance was only worse than that of MIDER. However, MIDER achieved FPR = 0 at the cost of TPR. Hence, our proposed method still offered great advantages. To intuitively explain the advantages of RWRNET, we show the network structure inferred by all methods (Figure 2). The first network in the figure is the true network structure, the second network is the network structure inferred by RWRNET, and the other networks are the network structures inferred by comparison method. The figure shows that the network structure inferred by CLR, RRMRNET, ARACNE, and MIDER was the closest to the true network, whereas the results obtained by MRNET, PCA-CMI, and MI3 contained considerable redundant control relationships. RWRNET missed X1–X8 and incorrectly linked X8 to other genes, similar to the other methods. Only MI3, MIDER and PCA-CMI were able to predict X1–X8. However, MIDER missed X3–X4 and X5–X6, MI3 and PCA-CMI introduced excessive redundant regulatory relationships. In summary, the proposed method showed excellent performance. Finally, by combining the performance of RWRNET in these two datasets, we learned that RWRNET is suitable for special networks.

**TABLE 4:** Comparison of the different methods’ performances in the reaction chain with eight species dataset.

	TP	FP	TPR	FPR	PPV	ACC
CLR	6	1	0.857	0.048	0.857	0.929
ARACNE	6	1	0.857	0.048	0.857	0.929
MRNET	6	9	0.857	0.429	0.4	0.643
MI3	2	11	0.286	0.524	0.154	0.429
MIDER	5	0	0.714	0	1	0.929
MRMSn	–	–	–	–	–	–
RRMRNET	6	2	0.857	0.095	0.75	0.893
PCA-CMI	6	16	0.857	0.762	0.273	0.393
RWRNET	6	1	0.857	0.048	0.857	0.929

### Results of the DREAM3 Challenge Network

To demonstrate that the proposed method can be used to infer GRNs from simulated dataset, we tested it in DREAM3. The DREAM3 Challenge Network is a version of the DREAM project that provides various gene expression datasets and corresponding golden networks to evaluate the performance of the inferred model. The gene expression dataset provided by DREAM3 is a simulation dataset. We used yeast gene expression data with a size of 10, 50, and 100 as the test datasets.

**FIGURE 3:**
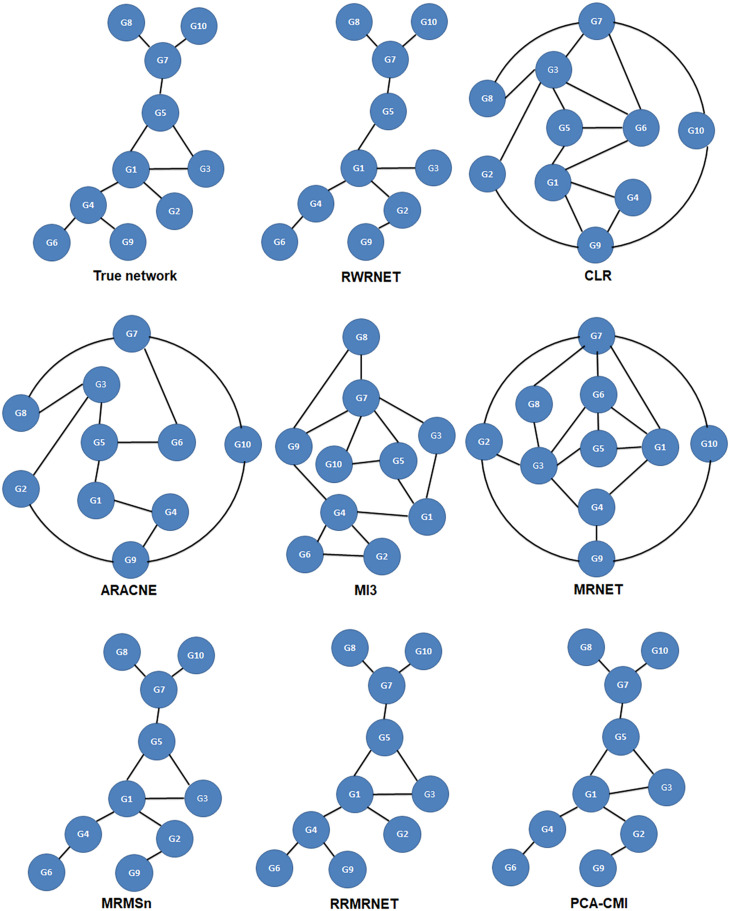
Comparison of the different methods in the Dream3-10 gene dataset.

First, we tested the proposed method in the yeast gene expression dataset with a size of 10. A comparative analysis of different methods is summarised in Table 5. RRMRNET had the best performance (PPV = 1, ACC = 1). MRMSn and PCA-CMI identified nine correct regulatory relationships (TP = 9), whereas RWRNET identified eight regulatory relationships only (TP = 8) and introduced a redundant regulatory relationship (FP = 1). To analyse visually the gap between RWRNET and other methods, we showed the network structure they inferred (Figure 3). The figure contains nine networks. The first network is a standard network, and the one on the right of the standard network is the network inferred by RWRNET. Like most other methods, RWRNET missed G4–G9 and predicted G2–G9 incorrectly probably because of noise in the data. Unfortunately, RWRNET also missed G3–G5. Similar to RWRNET, the network structure inferred by MI3 lost G3–G5 because MI3 cannot recognise the triangle relationship between G1, G3, and G5. Similarly, the loss of G3–G5 in our proposed method may have been caused by the complex network structures between G1, G3 and G5. Although RWRNET did not perform as well as the RRMRNET, MRMSn and PCA-CMI, it still performed well in terms of these four metrics compared with CLR, ARACNE, MRNET, and MI3.

**TABLE 5:** Comparison of the different methods’ performances in the Dream3-10 gene dataset.

	TP	FP	TPR	FPR	PPV	ACC
CLR	6	10	0.6	0.286	0.375	0.689
ARACNE	6	6	0.6	0.171	0.5	0.778
MRNET	6	12	0.6	0.343	0.333	0.644
MI3	8	6	0.8	0.171	0.571	0.822
MIDER	–	–	–	–	–	–
MRMSn	9	1	0.9	0.029	0.9	0.956
RRMRNET	10	0	1	0	1	1
PCA-CMI	9	1	0.9	0.029	0.9	0.956
RWRNET	8	1	0.8	0.029	0.889	0.933

We then tested the performance of the proposed method in the yeast gene expression dataset with a size of 50 (Table 6). The TPR of the proposed method was 0.377, whereas that of the others was between 0.052 and 0.494. RRMRNET was the only method that performed better than RWRNET in terms of TPR. The FPR of the proposed method was only 0.014, whereas the minimum FPR of the other methods was 0.015. The proposed method clearly identified correct regulatory relationships and avoided redundant regulatory relationships (TP = 29, FP = 16). In addition, the proposed method outperformed the other methods in all metrics, especially with an ACC of 0.948. In summary, the proposed method evidently performed better than the other methods.

**TABLE 6:** Comparison of the different methods’ performances in the Dream3-50 gene dataset.

	TP	FP	TPR	FPR	PPV	ACC
CLR	19	165	0.247	0.144	0.103	0.818
ARACNE	13	125	0.169	0.109	0.094	0.846
MRNET	21	215	0.273	0.187	0.089	0.779
MI3	21	68	0.273	0.059	0.236	0.899
MIDER	4	79	0.052	0.069	0.048	0.876
MRMSn	21	17	0.273	0.015	0.553	0.94
RRMRNET	38	56	0.494	0.049	0.404	0.922
PCA-CMI	25	19	0.325	0.017	0.568	0.942
RWRNET	29	16	0.377	0.014	0.644	0.948

Finally, we tested the performance of proposed method in the yeast gene expression dataset with a size of 100 (Table 7). The performance of RWRNET was superior to that of CLR, ARACNE, MRNET, MI3 and MIDER in all metrics. Compared with RRMRNET and PCA-CMI, RWRNET selected about 65 correct regulatory relationships (TP = 65) and introduced 50 redundant regulatory relationships (FP = 50). Although the TPR of RWRNET was not the highest (TPR = 0.392), its FPR was only 0.01. To sum up, the proposed method was considerably reduced the number of redundant regulatory relationships. Therefore, our method achieved the best performance in terms of PPV (PPV = 0.565) and ACC (ACC = 0.969).

**TABLE 7:** Comparison of the different methods’ performances in the Dream3-100 gene dataset.

	TP	FP	TPR	FPR	PPV	ACC
CLR	39	713	0.235	0.149	0.052	0.830
ARACNE	20	417	0.121	0.087	0.046	0.886
MRNET	49	984	0.295	0.206	0.047	0.778
MI3	27	165	0.163	0.035	0.141	0.939
MIDER	13	80	0.078	0.017	0.140	0.953
MRMSn	–	–	–	–	–	–
RRMRNET	92	238	0.554	0.05	0.28	0.937
PCA-CMI	70	64	0.422	0.013	0.522	0.968
RWRNET	65	50	0.392	0.01	0.565	0.969

In conclusion, RWRNET achieved a good performance in the DREAM3 challenge network dataset. The proposed method predicted as many correct regulatory relationships as possible while introducing the least redundant regulatory relationships. These features indicate that our method may be more advantageous than the other methods in inferring large-scale networks.

### Results of SOS Network in *E. coli*

Finally, we tested the performance of our method in the SOS network in *E. coli*. The SOS network is a signal pathway in the SOS DNA repair system, which has been experimentally confirmed and is often used to test the effectiveness of various methods in real networks. For gene expression data, we chose interference data, which were obtained through a series of transcription interference measurements.

The performance of all methods are analysed visually in Table 8. The performance of the proposed method was superior to that of the other methods, except for PCA-CMI in terms of ACC. In addition, the performance of the proposed method was the best in terms of PPV. At the same time, RWRNET had the best performance in terms of FPR, indicating that our method introduced fewer redundant regulatory relationships than the others. A real network usually has a complex network structure and close regulatory relationships. Thus, inferring a real network is difficult. However, compared with the other methods, the proposed method performed well in the SOS network, especially in identifying redundant regulatory relationships. This result demonstrated that our method can effectively reduce network complexity and thus it is suitable for inferring real networks.

**TABLE 8:** Comparison of the different methods’ performances in the SOS dataset.

	TP	FP	TPR	FPR	PPV	ACC
CLR	12	5	0.5	0.417	0.706	0.528
ARACNE	7	3	0.292	0.25	0.7	0.444
MRNET	17	6	0.708	0.5	0.739	0.639
MI3	9	5	0.375	0.417	0.643	0.444
MIDER	–	–	–	–	–	–
MRMSn	10	2	0.417	0.167	0.833	0.556
RRMRNET	10	2	0.417	0.167	0.833	0.556
PCA-CMI	19	3	0.92	0.25	0.84	0.778
RWRNET	15	1	0.625	0.083	0.938	0.722

## Discussion

In this article, we emphasised that combining local topology with global topology can be used to improve the accuracy of network inference. However, existing methods usually focus on local topology rather than on global topology. Given that RWR is a global search algorithm, we used it to obtain the global topology of the network. To confirm that RWR can be better applied to GRNs, we improved its three key elements. First, we constructed restart probability and initial probability vector on the basis of network characteristics and regulatory mechanisms to obtain the local topology structure. Second, we adopted the asymmetric ranking strategy in constructing the roaming network to reduce the complexity of regulatory relationships. Finally, we used IPC-MB to optimise the network structure. Thus, the proposed method (RWRNET) could theoretically infer accurate GRNs.

RWRNET was tested on simulated and real datasets. In simulated datasets, the proposed method achieved excellent performance. In the reaction chain with four species, the network structure inferred by RWRNET was exactly the same as the true network. In the reaction chain with eight species, the Dream3-50 gene dataset and the Dream3-100 gene dataset, RWRNET accomplished superior performance. In the Dream3-50 gene dataset, its PPV was 0.644 and ACC was 0.948, indicating that the proposed method had a relatively good effect. These results showed that combining local topology with global topology can effectively improve the accuracy of network inference. In real datasets, RWRNET also achieved satisfactory results. Under the premise that RWRNET obtained enough regulatory relationships (TP = 15), the redundant regulatory relationships it introduced were the least (FP = 1) possibly because the processing of roaming networks reduced the effects of complex regulatory relationships on RWR. Interestingly, RWRNET performed unsatisfactorily compared with the other network inference methods in the Dream3-10 gene dataset and SOS dataset. Two possible reasons can be offered: the complexity of network structure and the amount of noise in the data. In the Dream3-10 gene network, RWRNET missed G3–G5 because of the triangular relationship between G1, G3, and G5 that increased the complexity of the network structure. Moreover, the SOS network had a lot of noise that negatively affected the performance of the proposed method.

RWRNET was tested on networks of different sizes (i.e., different numbers of variables), containing 4, 8, 9, 10, 50, and 100 genes. The experimental results show that RWRNET achieved good performance on the six different scale networks. As shown in Tables 3–8, except for networks of sizes 9 and 10, the performance of RWRNET showed an upward trend with the increase in the number of genes (the number of variables) in the network. Especially in networks with sizes of 50 and 100, RWRNET achieved good results in terms of the PPV and ACC metrics. Thus, combining global topology with local topology can effectively improve the accuracy of network inference.

The performance of RWRNET was also compared with that of other gene network inference methods in terms of different evaluation metrics. Results showed that RWRNET performed better than the other methods for most datasets. In the Dream3-10 Gene Network and SOS Network datasets, RWRNET did not perform as well as PCA-CMI. Although the performance of RWRNET in these datasets was not satisfactory, it nevertheless considerably reduced the number of redundant regulatory relationships, indicating that the global topology relationships of the network can also improve the performance of network inference.

## Conclusion

In this study, we proposed a novel network inference method based on information theory and RWR. We improved the three key elements of RWR to infer GRNs by using the proposed method. Restart probability was calculated, initial probability vector was constructed to adapt to network characteristics and regulatory mechanisms as much as possible to capture the network topology accurately. Moreover, a roaming network construction algorithm based on asymmetric ranking was proposed. This algorithm effectively reduced the effects of complex regulatory relationships on RWR. Finally, the local topology was combined with the global topology through RWR to infer the network structure. IPC-MB was used to deal with isolated nodes and optimise the network structure. The proposed method was tested in six standard network datasets, and its performance was compared with that of eight state-of-the-art methods based on information theory. Experimental results confirmed that the proposed method can efficiently and accurately infer GRNs.

## Data Availability Statement

All datasets presented in this study are included in the article/supplementary material.

## Author Contributions

WL and XS implemented the experiments, analysed the result, and wrote the manuscript. LP and LZ analysed the result. HL and YJ provided the constructive discussions and revised the manuscript. All authors read and approved the final manuscript.

## Conflict of Interest

The authors declare that the research was conducted in the absence of any commercial or financial relationships that could be construed as a potential conflict of interest.

## Footnotes

1 https://github.com/Dam-1517/RWRNET

2 https://www.genome.jp/kegg/

3 http://dreamchallenges.org/project-list/

4 http://regulondb.ccg.unam.mx/index.jsp/
